# Serious challenges with bone cement in orthopedic operating rooms: an observational study from Alborz province, Iran

**DOI:** 10.1186/s13018-024-05442-z

**Published:** 2025-03-12

**Authors:** Leila Sadati, Rana Abjar, Salman Azarsina, Samaneh Doroudian, Fatemeh Tavakoli

**Affiliations:** 1 Department of Operating Room, School of Paramedical Sciences, Alborz University of Medical Sciences, Karaj, Iran; 2 Department of Orthopedics, School of Medicine, Shahid Madani Hospital, Alborz University Of Medical Sciences, Karaj, Iran; 3 Student Research Committee, School of Paramedical Sciences, Alborz University of Medical Sciences, Karaj, Iran; 4 Alzahra Hospital, Alborz University of Medical Sciences, Karaj, Iran

**Keywords:** Bone cement, Total knee arthroplasty, Chemical hazards, Operating room

## Abstract

**Background:**

Alongside the numerous advantages of arthroplasty surgery, the extensive complications associated with bone cement contact remain serious chemical hazards in the operating room. The present study aims to investigate the challenges of using bone cement in orthopedic operating rooms.

**Method:**

This is a cross-sectional study conducted from September 2023 to June 2024 with the aim of examining the physical facilities in orthopedic operating rooms and the performance of orthopedic surgical teams in adhering to standards related to the use of bone cement. The performance of 300 personnel working in orthopedic surgical teams in seven operating rooms was assessed. The data collection tools consisted of two checklists, consisting of 15 and 10 items, prepared based on the latest valid international guidelines. The collected data were analyzed using SPSS version 28.

**Results:**

Data analysis revealed that 14.2% of the operating rooms were in an unfavorable condition in terms of having facilities and physical amenities for the application of bone cement, while the remaining 85.8% had relatively favorable conditions. Regarding the average adherence to performance standards by surgical team members, 14.3% of participants were in an unfavorable condition, 78% were in a relatively favorable condition, and 7.7% were in a favorable condition.

**Conclusion:**

Considering the lack of protective facilities in operating rooms, attention to providing these facilities is essential. Also, based on a deficiency in adherence to some performance standards by surgical team members, training them and giving up-to-date guidelines is recommended.

## Background

Total knee arthroplasty (TKA) is one of the most common orthopedic surgeries, with its incidence increasing nearly by 100% in recent years [1]. This dramatic increase in surgical volume has direct implications for occupational safety, as it means proportionally higher exposure to bone cement among operating room personnel. Various factors contribute to the rise in TKA cases, with osteoarthritis prevalence being among the most significant, as nearly 89% of patients undergoing surgery exhibit severe osteoarthritis symptoms, including unbearable pain and walking difficulties [2, 3]. Another significant factor contributing to the prevalence of osteoarthritis and the increasing number of knee arthroplasty surgeries in recent years is obesity [2, 4, 5]. Studies have shown a strong association between the rising incidence of knee replacement surgeries and obesity prevalence in different countries [6]. The third factor driving this surgery is osteoporosis, with various reasons such as aging, improper diet, sedentary lifestyle, corticosteroid use, and hormonal disorders contributing to its increase [7]. In TKA method, the entire tibiofemoral joint is replaced with a prosthesis.

Fixation of the prosthesis can be done through three methods: cemented fixation, cementless fixation, and hybrid fixation. In the hybrid fixation, the tibial side is cemented, and the femoral side is cementless [8]. Bone cement, or Poly methylmethacrylic acid (PMMA), is a polymer used for joint fixation. Its precursor consists of two components, liquid and powder, which are mixed and form a doughy substance a few minutes before cementation [9]. This polymer has widespread applications in orthopedic surgeries, ophthalmology, plastic surgery, and dentistry, with studies showing an annual increase in demand for it since 2005, reaching 3.5% [10, 11]. This polymer, during the mixing process, releases methyl methacrylate vapors which, at certain concentrations, may present occupational exposure concerns for healthcare workers [12, 13]. Studies have documented various effects of prolonged exposure, ranging from mild irritation to more significant health implications when proper safety measures are not implemented [14]. The use of bone cement presents two distinct categories of health concerns. First, patients may experience Bone Cement Implantation Syndrome (BCIS), a well-documented surgical complication with potentially serious cardiovascular effects [15]. Separately, healthcare workers face occupational exposure risks during cement mixing and application, with documented effects including eye irritation, respiratory tract irritation, and increased mucus secretion [12]. These occupational hazards, which are the focus of this study, require specific preventive measures and safety protocols.

Other studies have shown that skin contact with bone cement can lead to contact dermatitis, numbness of fingertips, and nasal discharge. These adverse effects can be prevented by proper ventilation and the use of masks containing carbon filters, face shields, goggles, and closed mixing equipment [10, 14, 15] . Also, the result of a study has shown that female orthopedic surgeons with high exposure to bone cement had a 3.97 times higher prevalence of breast cancer compared to other women [16].

Given these documented health risks and preventive measures, performance standards have been established as formalized protocols and procedures that surgical team members must follow when handling bone cement. These standards, codified in Occupational Safety and Health Administration (OSHA) and Association of periOperative Registered Nurses (AORN) guidelines, systematically address each risk factor by mandating specific behaviors and practices. They transform the known preventive measures into actionable requirements, ensuring consistent implementation of safety protocols across all aspects of bone cement handling.

Based on this, guidelines published by various organizations and associations, including OSHA and AORN, provide recommendations for preventing these occupational hazards related to chemical exposures. These guidelines specify detailed technical requirements for operating rooms where bone cement is used. Environmental controls include maintaining specific air replacement rates and laminar airflow ventilation systems. Personal protective equipment requirements specify the use of specialized items such as Neoprene gloves for cement handling and masks with carbon filters. The guidelines also mandate the use of closed mixing systems for cement preparation to minimize vapor exposure. Each of these requirements has been established based on evidence of their effectiveness in reducing occupational exposure risks [19, 20]. The positive impact of considering these recommendations is evident in numerous studies [21, 22].

Alongside the increasing trend of joint replacement surgeries in operating rooms, despite serious recommendations related to preventing the hazards of exposure to this hazardous chemical substance, there is evidence indicating significant weaknesses in adhering to these standards in the physical structure and performance of surgical team members.

Considering the numerous studies on the hazards of inhaling and contacting bone cement, as well as the established standards for adhering to principles in the cement application process, it seems crucial to pay attention to these standards to prevent endangering the health of both staff and patients. Therefore, this study aimed to investigate the status of facilities, physical amenities, and the performance of orthopedic surgical teams compared to the standards developed for the use of bone cement in orthopedic operating rooms in Alborz province.

## Methods

This descriptive, analytical study was conducted cross-sectionally from 2023 to 2024 with the aim of assessing the status of facilities, physical amenities, and the performance of orthopedic surgical teams compared to the standards developed for the use of bone cement in orthopedic operating rooms in Alborz province.

### Sampling

In the current study, the physical structure of operating rooms and the performance of orthopedic surgical team members in seven hospitals in Alborz province were evaluated in terms of adherence to standards in the process of bone cement application. Sampling was conducted using the convenience sampling method based on criteria for inclusion in the study, including work experience in the orthopedic team and participation in joint replacement surgeries as a sterile member of the surgical team, as well as the willingness to participate in the study among the staff working in the operating rooms. Based on these criteria, 300 members of the surgical team participated in this study.

### Data collection tools

The data collection tools included a demographic questionnaire, a 15-item checklist related to performance standards of orthopedic surgical team members in the process of using bone cement, and a 10-item checklist related to physical standards and environmental facilities of orthopedic operating rooms in this regard, prepared based on the latest valid global guidelines. The demographic questionnaire included the name of the hospital, gender, marital status, educational background, employment status, tenure in the operating room, and experience in knee joint replacement surgery.

In the checklist sections, each question was scored one point for “yes” and zero points for “no” answers. For the performance standards checklist, which contained 15 items assessing adherence to safety protocols and procedures, scores ranged from zero to fifteen. These scores were then categorized into three levels based on established benchmarks: scores of 5 or lower were classified as undesirable, indicating significant gaps in safety protocol adherence; scores between 6 and 10 were deemed relatively desirable, suggesting moderate compliance with safety standards; and scores above 10 were classified as desirable, representing strong adherence to safety protocols.

Similarly, the physical standards checklist, comprising 10 items evaluating facility conditions and available safety equipment, was scored from zero to ten. These scores were also categorized into three levels: scores of 3 or lower were classified as undesirable, indicating substantial deficiencies in physical facilities and safety equipment; scores between 4 and 6 were considered relatively desirable, suggesting adequate but not optimal conditions; and scores above 6 were classified as desirable, representing well-equipped facilities meeting most safety requirements.

The validity of the tool used was confirmed through content validity ratio (CVR) = 0.82 and content validity index (CVI) = 0.79, obtained through a survey of 7 experts in the field, including orthopedic specialists, occupational health, and operating room professionals. The reliability of the checklist was calculated using inter-rater agreement, simultaneous evaluation of two evaluators, and calculation of the kappa coefficient, which was found to be 0.86.

### Data analysis

Data analysis was conducted by two researchers who were present in the operating rooms under study. They used an observational method to complete the checklist related to the standard of the physical environment concerning the structure and environmental facilities of the operating room. Additionally, after obtaining permission from the surgical team members, they attended knee replacement surgeries and, while completing the demographic questionnaire, recorded the performance of sterile orthopedic surgical team members in adhering to the standards related to the use of bone cement using a specific checklist. The collected data were then analyzed using SPSS version 28 software.

The data analysis section used statistical indicators of number, frequency percentage, mean, and standard deviation to describe the variables. In the inferential section, Friedman’s ranking tests (for ranking questions), independent T-test, and analysis of variance (ANOVA) were used to compare the means of performance and physical standards across demographic variables. Pearson correlation test was employed to examine the relationship between performance standards and background variables such as age and work experience. The maximum level of significance (alpha) for hypothesis testing was considered to be 0.05 (*p* < 0.05).

## Results

Based on the data analysis, 79.7% of the participants were male (239 individuals), and 20% were female. Among the participants, 66.3% (199 individuals) were surgical technologists, 25% were orthopedic surgeons (75 individuals), 4.7% were surgical technicians (14 individuals), and 4% were orthopedic assistants (12 individuals). The average age of the respondents was 46.40 years, with the youngest and oldest ages being 22 and 67 years, respectively. The average work experience in the operating room was 15.67 years, ranging from a minimum of one year to a maximum of 35 years. The average work experience in orthopedic surgery was 10.88 years, ranging from less than one year to a maximum of 35 years. Regarding marital status, the majority of respondents (72%) were married, and 28% were single. For clarification, surgical technologists in this context are healthcare professionals with a bachelor’s degree in operating room technology who assist surgeons and manage surgical equipment, while surgical technicians have a two-year associate degree with more limited scope of practice. Both roles are integral to the surgical team but differ in their level of training and responsibilities.

The results of the study regarding the physical standards and environmental facilities of the operating rooms under investigation showed that one of the operating rooms (14.2%) was in an undesirable condition, while six operating rooms (85.8%) were in a relatively desirable condition, and none of the operating rooms were in a desirable condition. In assessing the status of facilities and amenities available in various items of the evaluation checklist, according to the findings of the Friedman test and considering the calculated Chi-square value of 120.212, with a significance level less than 0.001 (*p* < 0.001), there was a significant difference in the ranks of the items with at least 95% confidence. Based on this, the three items that obtained the highest rank were items 1 to 3, and the three items with the lowest rank were items 8 to 10 (Table 1).

**Table 1:** Ranking the items related to physical standards and environmental facilities

Rank	Items	Average Rank	Percentage of Yes Responses
1	Is the operating room equipped with a powder and foam fire extinguishing system?	8.25	100
2	Is there a moisture meter for humidity measurement in the operating room?	8.23	100
3	Is there a thermometer for temperature monitoring in the operating room?	8.10	97
4	Is there a protective shield for covering the face and eye protection during the use of bone cement in the operating room?	7.47	84
5	Is the air in the operating room replaced at least 19 times per hour?	4.98	35
6	Is there a standard protocol regarding precautionary principles when working with bone cement in the operating room?	4.80	31
7	Is the operating room equipped with a laminar airflow ventilation system?	3.42	3
8	Are Neoprene gloves available for preparing bone cement in the operating room?	3.25	0
9	Is the operating room equipped with a closed system for mixing liquid and powder components of bone cement?	3.25	0
10	Is there a special mask with a carbon filter for personnel to use during cementation in the operating room?	3.25	0

Chi square = 2120.59; df = 9; *p* < 0.001

In the investigation of the surgery team member performance standards, the results of the Friedman ranking test showed a significant difference in the average rating of the items (*p* < 0.05). According to the results, the highest standards were related to the item “Do the surgical team members remove their contact lenses before facing the cement vapors?“, which was observed in 100% of cases. Items 13 to 15 (the use of a mask with a carbon filter by sterile team members, the use of closed system equipment to combine two liquid and powder components, and the use of Neoprene gloves during bone cement contact) were not observed in any operating room (Table 2).

**Table 2:** Ranking the items related to performance standards of surgical team members while using bone cement

Rank	Items	Average Rank	Percentage of Yes Responses
1	Do surgical team members remove their contact lenses before exposure to cement fumes?	11.72	100
2	Do sterile members wear more than one pair of gloves?	11.27	94
3	Is cement waste disposed of as hazardous waste?	11.00	90
4	Is the non-use of electrocautery during cementation observed?	10.60	85
5	Is supervision over the disposal of cement waste as hazardous waste conducted?	9.72	73
6	Is the powder and foam fire extinguishing system in the operating room monitored by personnel?	9.35	68
7	Is the room temperature set between 21 to 23 degrees Celsius?	9.32	68
8	Do sterile team members use protective glasses?	9.07	64
9	Has the protocol regarding standard principles in using bone cement been studied?	7.00	37
10	Is the room humidity adjusted between 36 to 55%?	6.70	33
11	Are gloves immediately replaced after cementation?	6.22	26
12	Is suction used to remove fumes during open mixing?	5.27	14
13	Do sterile team members use masks with carbon filters?	4.25	0
14	Is closed system equipment used to mix liquid and powder components?	4.25	0
15	Are Neoprene gloves used during cementation?	4.25	0

Chi Square = 2202.64; df = 14; *p* < 0.001

In Tables 3, 4 and 5, the relationship between background variables (job groups, marital status, age, and work experience) was tested in compliance with performance standards.

**Table 3:** Comparison of the performance standards according to the status of job groups

Variable	Job-groups				F	*P*
	Orthopedy (*n* = 75)	Orthopedy -assistance (*n* = 12)	Surgical technician (*n* = 14)	Surgical technologist (*n* = 199)		
Performants-standards	49.33	37.78	46.19	51.29	5.09	0.002

The results of the analysis of variance in Table 3 showed that there was a significant difference in the level of performance standards compliance between job groups (*p* < 0.05). The post hoc test revealed that the highest level of compliance with performance standards was related to surgical technologists, with 51.29%, and the lowest level of compliance with the performance standards was related to the orthopedic assistant group, with a value of 37.78, which was significantly lower than surgical technologists and orthopedic specialists (*p* < 0.05).

**Table 4:** Comparison of the average performance standards according to marital status

Variable	marital status		Mean difference	t	*p*
	Married (*n* = 216)	Single (*n* = 84)			
Performants-standards	52.28	44.21	8.08	5.16	< 0.001

The results of the independent t-test in Table 4 showed that a significant difference was observed in the level of performance standards compliance between single and married people (*p* < 0.05). The average compliance with performance standards among single people was 44.21, which was significantly lower than the average of married people, with an average of 52.28%.

**Table 5:** Examining the relationship between performance standards and the variables of age and work experience in orthopedics

Variables		Correlation Coefficient	*p*
Performance standards	Age	0.085	0.143
	Work experience in operating room	0.133	0.021
	Work experience in orthopedic field	-0.060	0.301

The results of Pearson’s correlation test in Table 5 showed that there was no relationship between age and experience of orthopedic surgery filed with performance standards (*p* < 0.05). Also, the findings showed that there was a significant positive relationship between work experience in the operating room and compliance with performance standards (*p* < 0.05). This means that increasing work experience in the operating room improves performance standards.

## Discussion

The results of the current study aimed to investigate the adherence to physical and performance standards regarding the use of bone cement during knee arthroplasty surgeries in selected hospitals in Alborz province showed that in terms of adherence to physical standards and environmental facilities, one operating room (14.2%) was in an undesirable condition, and six cases of operating rooms (85.8%) were in a relatively desirable condition. In assessing the adherence to performance standards by the surgical team members, 14.3% of participants were in an undesirable condition, 78% were in a relatively desirable condition, and only 7.7% were in a desirable condition and adhered to the principles of standard care.

In examining the items related to performance standards, the highest score belonged to the items of having a powder and foam capsule for extinguishing fires caused by cement, and the presence of a humidity meter and thermometer, which play a key role in managing and preventing fires in operating rooms. Sibia et al. in 2016, while mentioning the statistics of 600 annual fires in operating rooms, report a case of fire caused by the use of an electrocautery device alongside bone cement in joint replacement surgery and emphasize the importance of having appropriate fire extinguishing equipment and training staff in its use in cases of fire [23]. Leonovicz O and colleagues also shared their experience of a fire during a joint replacement surgery. According to them, a fire occurred when bone cement was removed with a Freer elevator, and they could not extinguish it using a moist sponge. Therefore, awareness of fire extinguishing methods and the availability of sufficient facilities in these operating rooms are essential. Researchers recommend preventing the accumulation of excess cement and collecting vapors from polymethyl methacrylate with proper ventilation to reduce the risk of fire [24]. More than three decades ago, Limonene stated that fires resulting from cement combustion are not controlled with water and require powder and foam capsules [25].

On the other hand, the lowest score obtained in the checklist assessing the physical and structural standards related to operating room design was due to the absence of protective equipment and the lack of a closed vacuum mixing system. Patel and colleagues 2022 addressed specifically the occupational hazards to orthopedic surgeons related to polymethyl methacrylate in an article. In this study, the importance of using a vacuum mixing system is evident when the concentration of bone cement vapors in manual mixing is 17 ppm, compared to 4 ppm in vacuum mixing. In manual mixing, the likelihood of reaching toxic vapor concentrations is very high [26]. Additionally, Dagci et al. in 2018, through a systematic review of articles related to bone cement over the past 20 years, demonstrated that vacuum mixing is one of the key methods to protect the health of orthopedic operating room personnel in contact with cement. They mention standard ventilation and laminar systems as protective measures against the hazards of inhaling toxic cement gases [27]. One of the protective equipment whose absence in the operating rooms was a serious weakness was Neoprene gloves resistant to bone cement penetration. In various studies, in addition to the importance of the number of glove layers and the immediate removal of the last layer after cementing, the material of the gloves has also been emphasized [28]. The study by Thomas S. and colleagues also demonstrates that compared to Vinyl and Latex gloves, Neoprene gloves are resistant to cement penetration and are impermeable to polymethyl methacrylate for up to 25 min [29]. None of the operating rooms had this type of gloves, which increases the likelihood of contact dermatitis and long-term adverse effects of contact with bone cement. Additionally, in the assessment of the physical and structural standards of the operating rooms, masks with carbon filters were not available. Compton J. and colleagues in 2020, through laboratory studies, introduced carbon filter masks as the best means of reducing inhalation risks of methyl methacrylate. These masks prevent the absorption and passage of all toxic vapors in practice [21].

As previously mentioned, in assessing the adherence to performance standards by the surgical team members, 14.3% of participants were in an undesirable condition, 78% were in a relatively desirable condition, and only 7.7% were in a desirable condition and adhered to the principles of standard care. In examining the items related to performance structure, the highest score obtained belonged to the items related to the removal of contact lenses during the use of cement by surgical team members, avoiding the use of cautery until the bone cement is completely dry, separate disposal of waste, and wearing double gloves. Green G. and colleagues in 2011 highlighted this issue and warned of the possibility of corneal irritation and injury when contact lenses come into contact with cement vapors [30]. Eye injuries have been highlighted in numerous studies, including Kakazu et al. and Mohan Kumar et al. [17, 31].

In contrast, the items that received the lowest scores in the evaluation of the surgical team’s performance checklist included items 8 to 10, which were not adhered to due to the lack of necessary facilities and equipment. Following these three items, not using suction to reduce the volume of vapors emitted was one of the issues that, unfortunately, was only adhered to in 14% of cases, and standard attention was not paid to the inhalation hazards of bone cement. Kumar et al. in 2020 emphasized the use of suction in reducing toxic vapors emitted by bone cement [17]. Additionally, immediate glove change after completing the work is also a serious recommendation in guidelines related to working with bone cement, which has been emphasized in various studies but was only performed in 26% of cases [32, 33].

In examining the relationship between demographic characteristics and adherence to performance standards, the study results showed that among job groups, surgical technologists had the highest mean adherence to performance standards at 51.3%, followed by orthopedic surgeons with a mean of 49.3%, and the lowest mean adherence to performance standards was among orthopedic assistants at 37.8%. This could be related to the higher experience and more extensive training of operating room specialists in surgical nursing care. Additionally, independent group T-test analysis comparing marital status and adherence to performance standards showed a significant difference between single and married individuals (*p* < 0.05), with married individuals having a higher mean adherence to performance standards. This correlation might be related to factors such as greater average work experience in this demographic group, though further research would be needed to establish any causal relationships. Furthermore, Pearson correlation analysis indicated a significant positive relationship between work experience in the operating room and adherence to performance standards (*p* < 0.05), which also supports the arguments presented in the two aforementioned cases and underscores the importance of training young and less-experienced personnel. Manouchehri et al. in 2015, emphasized the importance of work experience and expertise in enhancing the knowledge and skills of young nurses [34]. The study by Mescour et al. (2021) also demonstrates that work experience plays a significant role in professional competence and delivering high-quality care to patients [35].

## Conclusions

The conclusion drawn from the present study indicates a wide range of structural issues and deficiencies in equipment and facilities in the physical standards dimension. These deficiencies are important factors in occupational risk control when dealing with bone cement. The significance of this issue becomes greater when physical standards not only affect the health of healthcare staff but also impact the health and rapid improvement of patients. Moreover, in the assessment of performance standards, the study’s results suggest a lack of adherence to vital principles in preventing risks associated with the use of bone cement. This could lead to irreparable damage to surgical team members. Therefore, effective and relevant training in operating room issues is imperative. Staff awareness in the operating room yields results when personal protective equipment and physical standards of the rooms adhere to global principles, as both issues together will be compelling. Hence, it is hoped that with proper planning in both areas, progress will be made.

## Data Availability

No datasets were generated or analysed during the current study.

## Abbreviations

TKA: Total knee arthroplasty
PMMA: Poly methylmethacrylic acid
BCIS: Bone cement implantation syndrome
OSHA: Occupational Safety and Health Administration
AORN: Association of periOperative Registered Nurses
CVR: Validity ratio
CVI: Content validity index
ANOVA: Analysis of variance
